# The effect of *Ganqichengpi* syndrome diarrhea and *Tongxie Yaofang* intervention on the intestinal mucosal microbiota and neurochemical substances

**DOI:** 10.3389/fcimb.2025.1651581

**Published:** 2025-09-09

**Authors:** Chengxing Long, Yan Liu, Zizhen Yu, Jialin Liu

**Affiliations:** ^1^ Hunan University of Humanities, Science and Technology, Loudi, China; ^2^ Hunan Provincial Veterans Administration Hospital, Changsha, China

**Keywords:** *Tongxie Yaofang* prescription, *Ganqichengpi* syndrome diarrhea, intestinal mucosa, microbiota, neurochemical substances

## Abstract

**Objective:**

To explore the regulatory effect of *Tongxie Yaofang* prescription on intestinal mucosal microbiota in mice with *Ganqichengpi* syndrome diarrhea by 16S rRNA high-throughput sequencing.

**Methods:**

*Ganqichengpi* syndrome diarrhea model was replicated via the *Folium senna* extract combined with restraint and tail pinch stress. After the model was successfully replicated, the treatment was carried out with the *Tongxie Yaofang*. Once the modeling and treatment experiments were completed, the levels of 5-hydroxytryptamine (5-HT), vasoactive intestinal peptide (VIP), and brain-derived neurotrophic factor (BDNF) in serum were determined by enzyme-linked immunosorbent assay. The pathological changes of liver tissues were detected by HE staining technology, and the characteristics of intestinal mucosal microbiota were analyzed by 16S rRNA gene high-throughput sequencing technology.

**Results:**

The results showed that modeling significantly increased the levels of VIP and BDNF, and significantly decreased the level of 5-HT. After the intervention of *Tongxie Yaofang*, the level of BDNF remained high, but it had no significant effect on 5-HT and VIP. The results of the relative abundance of microbiota showed that Bacillota was the main microbiota in the intestinal mucosa and occupied an absolute dominant position. At the phylum level, the modeling of *Ganqichengpi* syndrome diarrhea increased the relative abundance of Bacillota, significantly raising the ratio of Bacillota to Bacteroides. After treatment with *Tongxie Yaofang*, they basically returned to the normal group level. At the genus level, *Clostridiaceae Candidatus*, *Lactobacillus*, and *Streptococcus* were the top three dominant genera. Modeling reduced the relative abundance of *Lactobacillus* and increased the relative abundance of *Clostridiaceae Candidatus*. After the treatment with *Tongxie Yaofang*, the relative abundance of *Lactobacillus* increased, while *Clostridiaceae Candidatus* and *Streptococcus* decreased. The LEfSe analysis results indicated that *Lactobacillus* was the biomarker genus in the normal group in the modeling experiments. Under the condition of an LDA threshold of 4, no biomarker genera were found in each group in the treatment experiment, but the relative abundance of *Lactobacillus* significantly increased in the treatment group.

**Conclusion:**

The therapeutic effect of *Tongxie Yaofang* was achieved by altering the abundance of *Lactobacillus*, the dominant microbiota in the intestinal mucosa, through three possible pathways.

## 1 Introduction

Modern life elements such as a fast-paced daily routine, a depressing working atmosphere, complex social networks, and irregular dietary habits have made patients with *Ganqichengpi* syndrome diarrhea very common in current clinical practice (Liu et al., 2019). The intestinal characteristics of a high proportion of Bacteroides and Prevotella in patients with the *Ganqichengpi* syndrome diarrhea all suggest that intestinal dysbacteriosis may be an influencing factor of *Ganqichengpi* syndrome diarrhea (Yuan et al., 2020a). Animal experiments have found that the modeling of *Ganqichengpi* syndrome diarrhea has changed the activity, number and structure of the intestinal microbiota in mice (Tang et al., 2020a, 2020b; Yuan et al., 2020b).

The intestinal mucosa is the largest contact surface between the internal environment of the animal body and substances in the intestinal lumen. Besides the function of absorbing nutrients, it also has the barrier function of effectively preventing pathogenic bacteria and solid antigens in the intestinal tract from invading the body (Long et al., 2018). At present, it is believed that various pathogenic bacterial infections, intestinal cavity diseases, inflammatory bowel disease, etc. are all related to intestinal mucosal immunity (Merga et al., 2014). Under normal circumstances, a healthy immune system and intestinal microbiota reinforce each other and jointly promote the host health (Guo et al., 2025). On the contrary, the changes in the intestinal microbiota caused by diarrhea can affect mucosal integrity and intestinal homeostasis (Jia et al., 2024).

Traditional Chinese medicine is rich in chemical components and has a wide range of pharmacological effects, which can directly or indirectly regulate the intestinal microbiota. The *Tongxie Yaofang* is a classic and renowned prescription for treating *Ganqichengpi* syndrome diarrhea (Tang et al., 2020b). Previous studies have shown that *Tongxie Yaofang* can regulate the abundance of intestinal microbiota, improve intestinal permeability, regulate the biocustomized resistance B/E value, and enhance the intestinal mucosal barrier function to treat diarrhea (Tang et al., 2020b; Liu, 2022; Zhang et al., 2022; Jia et al., 2024). However, the specific biomarkers for diagnosis and therapeutic are still unclear.

In this study, we analyzed the diversity and abundance characteristics of the intestinal mucosal microbiota in mice after the modeling of *Ganqichengpi* syndrome diarrhea and treatment with *Tongxie Yaofang* prescription using second-generation high-throughput sequencing technology from the perspective of intestinal mucosa, providing a more solid theoretical support for the treatment of diarrhea and related intestinal diseases with traditional Chinese medicine compound prescriptions.

## 2 Materials and methods

### 2.1 Materials

#### 2.1.1 Animals and feed

SPF-grade healthy male Kunming mice, with a body weight 20 ± 2 g were purchased by Hunan Slaccas Jingda Laboratory Animal Company. The production license for experimental animals was [SCXK (Xiang) 2019-0004]. All experimental animals were accompanied by quality certificates (No: 430727231100717725). The entire experiment was conducted in accordance with the standardized operation procedures approved by the Laboratory Animal Ethics Committee of Hunan University of Chinese Medicine (Approval Document Number: LL2023032901). The animal breeding adopts the standard experimental feed provided by the supplier. This product has been tested and meets the requirements of cleanliness and hygiene as well as no pollution.

#### 2.1.2 Experimental environment

The experiment was conducted in the ordinary-level laboratory animal room of the Laboratory Animal Center of Hunan University of Chinese Medicine. This venue has the following environmental conditions: The environment is clean and has low noise. Ventilation facilities and light-proof measures are all in place. The indoor temperature is maintained between 23°C and 25°C, and the relative humidity is controlled within the range of 50% to 70%. The license number for the use of experimental animals held by the experimental institution is SYXK (Xiang) 2019-0009.

#### 2.1.3 Drug preparation and kits

*Folium senna* was produced in Guangxi (batch number 2005302) and purchased from the First Affiliated Hospital of Hunan University of Chinese Medicine. 5-HT, VIP and BDNF ELISA kits (batch numbers: SU-B20715, SU-B20438 and SU-B20100 respectively) were provided by Quanzhou Konodi Biotechnology Co., Ltd.

The prescriptions of *Tongxie Yaofang* were purchased from the First Affiliated Hospital of Hunan University of Chinese Medicine based on the dosage of the original prescriptions in “Formulas of Traditonal Chinese Medicine”, including 9 g of Rhizoma *Atractylodis Macrocephalae* (Anhui, batch No. HQ23030601), 6 g of Radix *Paeoniae Alba* (Anhui, batch No. YH23030601), 4.5 g of Pericarpium *Citri Reticulatae* (Zhejiang, batch No. 221101), and 3 g of Radix *Saposhnikoviae* (Hebei, batch No. 230402). Soaked the above-mentioned medicinal materials in clean water for 30 minutes, and then decocted them in two batches. For the first decoction, the amount of water added should be sufficient to submerge the medicinal materials. After boiling over high heat, reduce to low heat and maintain a gentle boiling state for 30 minutes, then, filter out the medicinal liquid. The amount of water added for the second decoction should be halved, and the above decoction procedure should be repeated. The two decoctions were mixed and filtered through double-layer gauze. Then, the decoction was concentrated to the standard of 0.125g/ml of raw herbs, repackaged and stored in a 4°C refrigerator for later use (Liu et al., 2020).

### 2.2 Methods

#### 2.2.1 Animal grouping

Modeling period: 10 SPF-grade male Kunming mice were selected for the experiment. After 3 d adaptive feeding, tested mice were randomly divided into the control group (MC, n=5) and the model group (MM, n=5) by the random number table method. Treatment period: 15 SPF-grade male Kunming mice were divided into the control group (MCT, n=5) and the model group (n=10) by random number table method. After the successful replication of the model, tested mice in the model group were randomly divided into the traditional Chinese medicine intervention group (MTT, n=5) and the natural recovery group (MMT, n=5) by the random number table method.

#### 2.2.2 Model preparation

All the experimental mice were subjected to a 12-hour fasting treatment before gavage at 9:00 a.m. every day. The experimental mice in the model group were administered with 0.35 mL of *Folium senna* decoction, while the mice in the control group were treated with the same volume of distilled water. At 3:00 p.m., behavioral restriction intervention was implemented on the mice in the model group. The limb activities of the mice were restricted with a centrifuge tube fixation device, and mechanical stimulation was performed on the distal 1/3 of the tail with a special clip. Each intervention lasted for about 1 hour, while no stress treatment was given to the mice in the control group. After continuous intervention for 7 days, the mice in the model group showed extreme anger, wet and soft or loose stools, emotional excitement, roaring, aggressiveness, etc., indicating the successful construction of the model (Liu et al., 2020; Shao et al., 2024).

#### 2.2.3 Administration method and dosage

After the successful construction of the model, mice in the traditional Chinese medicine intervention group were gavaged with 0.35mL of *Tongxie Yaofang* prescription every day, while those in the control group and the natural recovery group were treated with 0.35mL of distilled water, twice a day and continuously for 5 days.

#### 2.2.4 Intestinal mucosa collection

The mice were sacrificed by cervical dislocation on a sterile operating table after the experiment was completed. Squeezed out the contents of the intestinal segments and cut the intestines longitudinally. Then, the residual contents on the intestinal wall were rinsed with normal saline, and the normal saline was absorbed with filter paper. The intestinal mucosa was scraped with a cover slip, weighed and labeled, and then placed in a centrifuge tube. All of the samples were kept in a refrigerator at -80°C for later use (Zhang et al., 2020).

#### 2.2.5 PCR amplification and sequencing

PCR amplification was performed using the universal primers of the bacterial 16S rRNA gene: The forward primer was 338F(5’ -ACTCCTACGGGAGGCAGCA-3 ‘), and the reverse primer was 806R(5’-GGACTACHVGGGTWTCTAAT-3’). The PCR response system was configured as follows: 5 μL of reaction buffer (5×), 5 μL of high GC buffer (5×), 0.25 μL of ABclonal DNA polymerase, 2 μL (10 mM) of dNTP mixture, 1 μL each of forward and reverse primers (10 uM), 2μL of DNA template and 8.75 μL of ultrapure water, the total volume was adjusted to 25 μL. The PCR amplification conditions were set as follows: Pre-denaturation at 98°C for 5 min; Subsequently, it enters a 25-cycle amplification stage. Each cycle includes denaturation at 98°C for 30 s, primer annealing at 52°C for 30 s, extension at 72°C for 45 s, with a final extended at 72°C for 5 min. Finally, the amplification product was stored at a low temperature of 12°C. The amplification products were purified and then subjected to double-ended sequencing analysis using the Illumina platform. The obtained data were processed by bioinformatics methods.

#### 2.2.6 Diversity analysis

To comprehensively evaluate the diversity characteristics of microbial communities, systematic analysis is usually conducted from two dimensions: alpha diversity and beta diversity. Alpha diversity mainly reflects the composition characteristics of the biological community within a specific biological territory, covering three core indicators: species richness, diversity and evenness. Among them, the Chao1 index and Observed species represent richness, the Shannon index and Simpson index represent diversity, and the Good’s coverage index represents coverage. The values of each index are positively correlated with the corresponding characteristics. Beta diversity index is mainly applied to evaluate the differences in species composition among different habitats. It is mainly achieved by means of principal coordinate analysis (PCoA) and non-measured multi-dimensional scaling analysis (NMDS). The core principle is to transform the high-dimensional sample similarity matrix into a low-dimensional visual map, thereby effectively presenting the ecological distance characteristics between samples and analyzing the differentiation laws of community structure (Long et al., 2023).

#### 2.2.7 Difference analysis and marker species

LEfSe analysis is the statistical methods by integrating of non-parametric Kruskal-Wallis and Wilcoxon rank sum tests, combined with the effect size evaluation system of linear discriminant analysis (LDA), focused on identifying the differentially characteristic bacterial species with statistical stability among different experimental groups, namely the marker species (Long et al., 2022). This method innovatively combines the robustness of non-parametric tests with the effect quantification of discriminant analysis, effectively enhancing the credibility of detecting differential microbial markers between groups.

#### 2.2.8 Histological observation

Fresh liver tissue of mice in each group was sliced and soaked in 10% formalin for 24 h, then gradient dehydrated and embedded in paraffin wax, after which the liver tissue blocks were cut into 3-5 μm thick sections and stained with hematoxylin and eosin (H&E) staining for morphological observation (Guo et al., 2025).

#### 2.2.9 Detection of neurotransmitters 5-HT, VIP and neurotrophic factor BDNF in serum

Whole blood specimens were collected via orbital blood extraction. Following collection, the blood samples were allowed to stand at room temperature for 2 hours to facilitate clotting. Subsequently, the samples were centrifuged at low temperature and high speed (4°C, 3000 rpm) for 20 minutes. The supernatant was collected for the determination of 5-HT, VIP, and BDNF levels. The concentrations of 5-HT, VIP, and BDNF in the serum were measured using enzyme-linked immunosorbent assay (ELISA). The specific operation was carried out in according to the instructions of the kit (Peng et al., 2023).

#### 2.2.10 Statistical analysis

IBM SPSS24.0 statistical software was used for the statistically analyzed. Measurement data obtained in each group was expressed as mean ± standard deviation. For normal data samples, One-way analysis of variance was used for the differences among multiple groups, and independent sample t-test was conducted between the two groups. The Mann-Whitney *U* test was used for non-normal distribution data. *P* < 0.05 indicates a significant difference, and *P* < 0.01 indicates an extremely significant difference (Long, 2025).

#### 2.2.11 Data storage

All raw data within this study has been deposited in the NCBI repository, accession number is PRJNA 1280487. (http://www.ncbi.nlm.nih.gov/bioproject/1280487)

## 3 Results

### 3.1 Pathological changes in the liver tissue of mice

**Figure 1:**
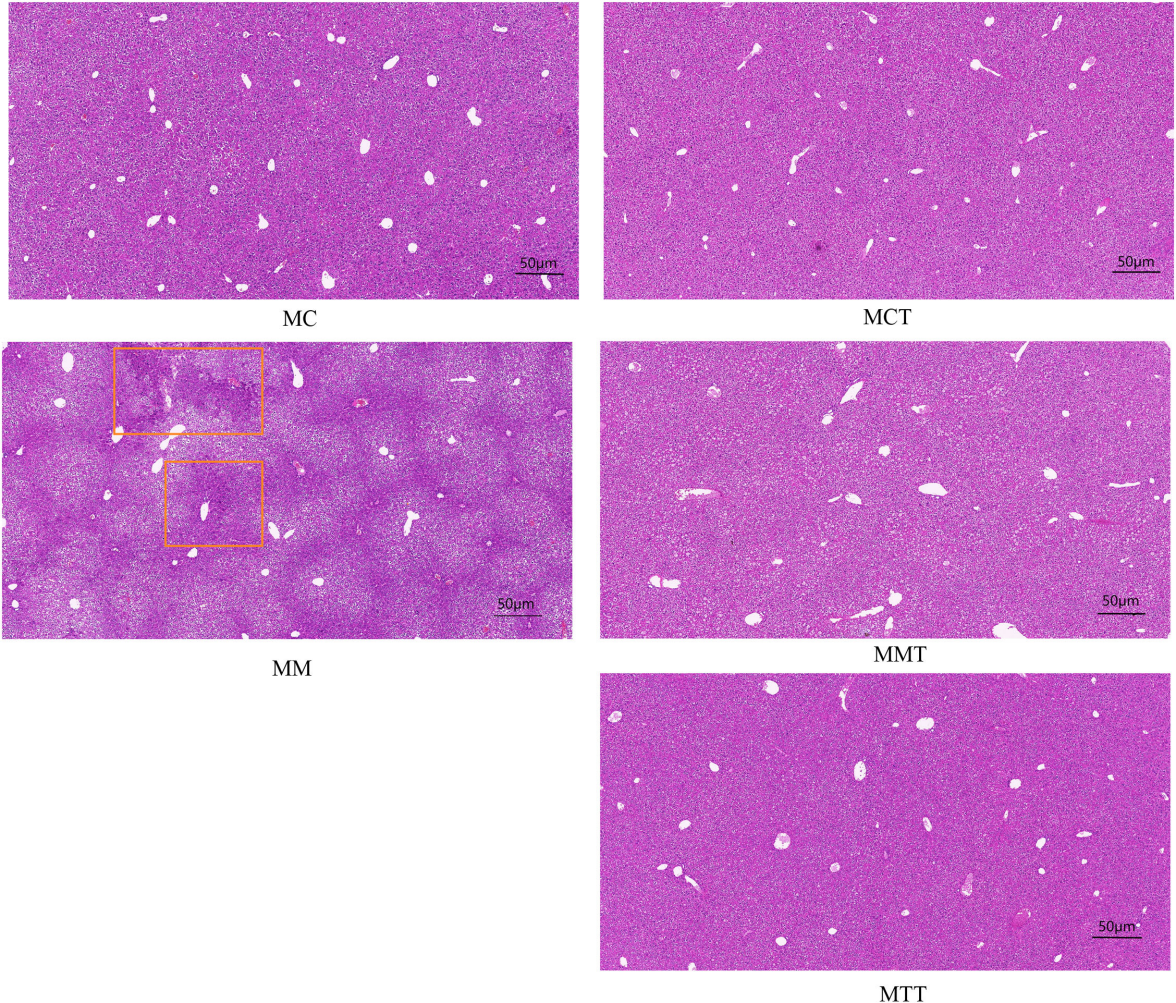
Pathological changes of liver tissue in mice during modeling experiments and treatment experiments. The red boxes indicated the pathology. MC stands for the control group, MM stands for the model group; MCT stands for the control group, MMT stands for the natural recovery group, MTT stands for the treatment group.

Liver tissue has many important physiological functions, including the intermediate metabolism of proteins, carbohydrates and fats, the synthesis of plasma proteins and important reproductive proteins, as well as the synthesis and secretion of bile (Zhang et al., 2025). Once liver tissue was damaged, it would have a serious impact on the metabolism of the entire body (Roberts and Rodger, 2012). As shown in **Figure 1**, the overall structure of the liver in MC was normal, and the morphology was relatively complete. The liver tissue cells in MM were hypertrophic, and vacuolation was aggravated. After the intervention with *Tongxie Yaofang*, the liver tissue was effectively restored to the level of normal mice.

### 3.1 The effect of *Ganqichengpi* syndrome diarrhea and *Tongxie Yaofang* intervention on intestinal mucosal microbiota sequences and OTU

**Figure 2:**
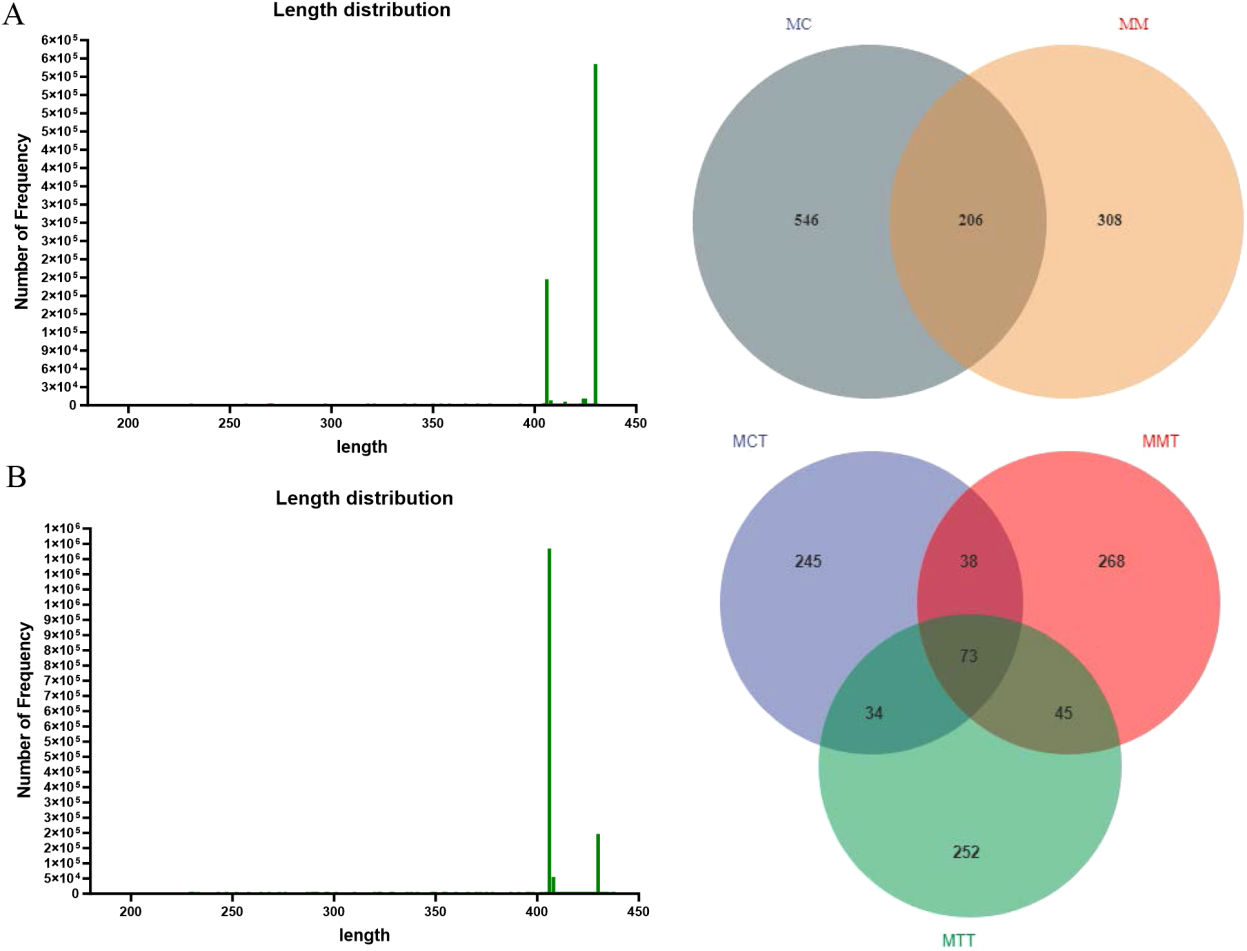
The effect of Ganqichengpi syndrome diarrhea and Tongxie Yaofang intervention on Intestinal mucosal microbiota sequences and OTU. **(A)** modeling experiment; **(B)** treatment experiment. MC stands for the control group, MM stands for the model group; MCT stands for the control group, MMT stands for the natural recovery group, MTT stands for the treatment group.

After quality control processing of the sequencing data, the lengths of the dominant sequences were mainly concentrated at 406 bp and 430 bp (**Figure 2**). The average value of the Good’s coverage index in each sample exceeded 0.9993, between 0.9986 and 0.9997, which could reflect the real situation of species in the community (**Table 1**). As shown in the Venn diagram of species, in the modeling experiment, a total of 1060 OTUs were obtained in the two groups. Among them, 752 OTUs in MC, accounting for 70.94%, and 514 OTUs in MM, accounting for 48.49%. The result indicated that the modeling of *Ganqichengpi* syndrome diarrhea reduced the number of OTUs of intestinal mucosal microbiota in mice. However, in the treatment experiment, the number of OTUs in the three group samples was basically similar, suggesting that treatment of *Tongxie Yaofang* had no significant effect on the number of OTUs of intestinal mucosal microbiota in mice.

**Table 1:** The coverage and alpha diversity indices of the intestinal mucosal microbiota in mice.

Experiment	Group	Chao1	Observed species	Simpson	Shannon	Goods coverage
Modeling	MC	274.81±52.45	243.58±47.06	0.7691±0.0690	3.3574±0.3947	0.9989±0.0003
	MM	175.32±14.73	152.92±12.78	0.7916±0.0340	2.9336±0.1650	0.9992±0.0001
Therapeutic	MCT	130.24±25.38	113.12±18.65	0.4255±0.2421	1.4205±0.7611	0.9994±0.0002
	MMT	137.04±26.07	121.26±24.12	0.4436±0.1749	1.5438±0.4999	0.9994±0.0001
	MTT	118.38±34.23	109.08±33.31	0.5680±0.2290	1.9654±0.8789	0.9996±0.0001

MC stands for the control group, MM stands for the model group; MCT stands for the control group, MMT stands for the natural recovery group, MTT stands for the treatment group. Compared with the control group.

### 3.2 The effect of *Ganqichengpi* syndrome diarrhea and *Tongxie Yaofang* intervention on intestinal mucosal microbiota diversity

#### 3.2.1 Intestinal mucosal microbiota alpha diversity characteristics

Alpha diversity encompasses a series of statistical indicators such as Chao1, Observed species, Shannon, and Simpson indices, mainly characterize the richness and diversity of ecological communities. The larger the index value, the higher their richness and diversity (Long et al., 2023).

In the modeling experiment, as indicated in **Table 1**, except for the Simpson index, all the diversity indices in MM were lower than those in MC, but there was no significant difference in the diversity indices between the two group samples (*P* > 0.05). In the treatment experiment, all diversity indices in MMT were higher than those in MCT (**Table 1**). After treatment with the *Tongxie Yaofang* prescription, the Chao1 index and Observed species index decreased, but the Simpson and Shannon indices increased.

#### 3.2.2 Intestinal mucosal microbiota beta diversity characteristics

**Figure 3:**
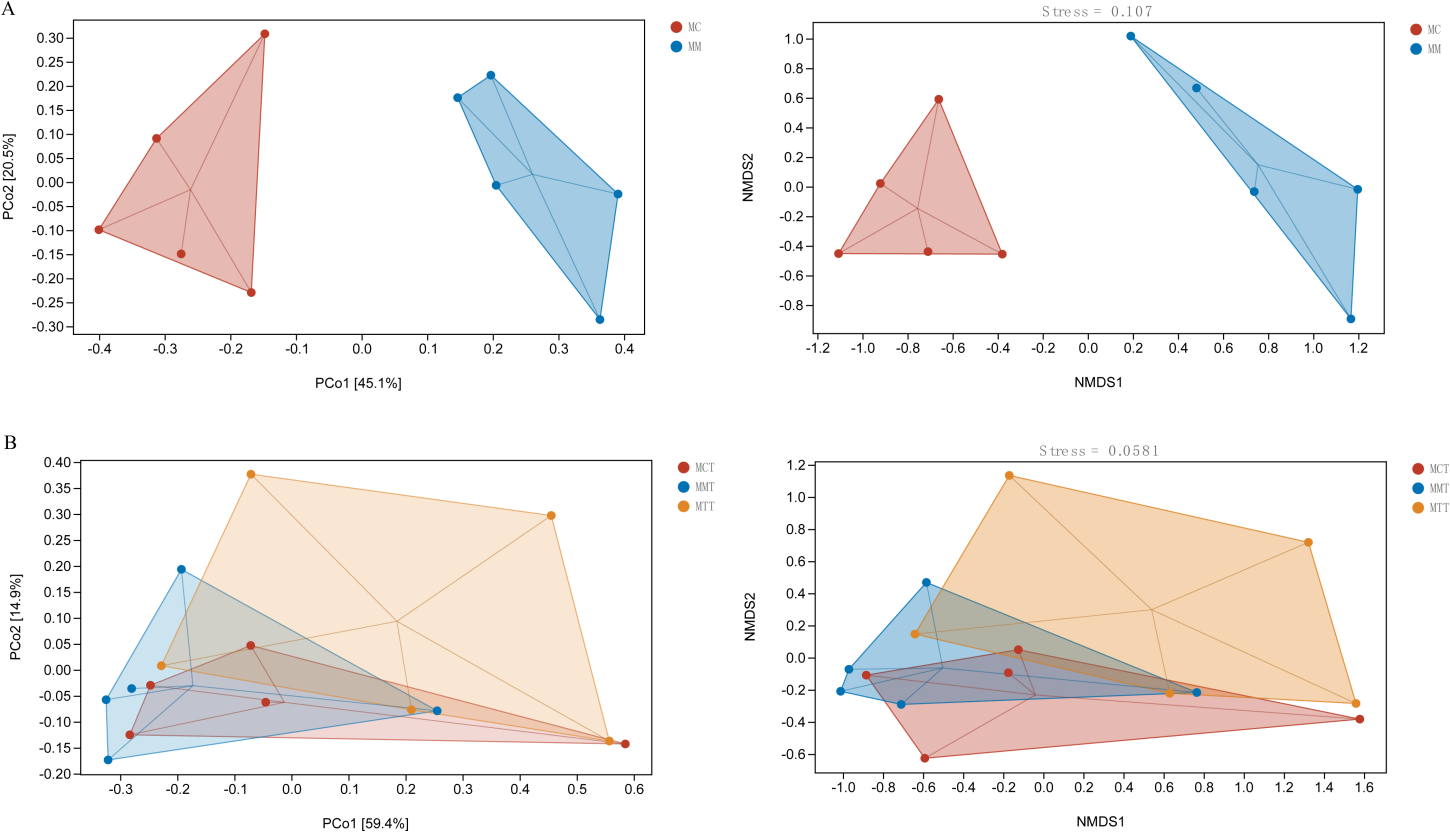
The effect of Ganqichengpi syndrome diarrhea and Tongxie Yaofang intervention on Intestinal mucosal microbiota beta diversity. **(A)** modeling experiment; **(B)** treatment experiment. MC stands for the control group, MM stands for the model group; MCT stands for the control group, MMT stands for the natural recovery group, MTT stands for the treatment group.

Beta diversity is mainly achieved through principal coordinate analysis (PCoA) and non-measured multidimensional scaling analysis (NMDS). PCoA analysis is one of the most classic unconstrained sorting analysis methods, which can retain the distance relationship of the original samples at the greatest extent. NMDS analysis is not affected by the sample distance value. For data with complex structures, the sorting results obtained by NMDS may be more stable. PCoA and NMDS analysis were performed on the three group samples using QIIME2 software. As indicated in **Figure 3**, in the modeling experiment, the samples between the normal group and the model group could be clearly separated, and the samples within the group were relatively dispersed. More interestingly, we could also draw the same conclusion from the treatment experiments. On the one hand, these results indicated that the differences within the group were relatively large. At the same time, it also showed that the effects of modeling and treatment on the bacterial structure in the intestinal mucosal mice were not obvious.

### 3.3 The effect of *Ganqichengpi* syndrome diarrhea and *Tongxie Yaofang* intervention on intestinal mucosal microbiota community composition

To explore the effects of *Ganqichengpi* syndrome diarrhea modeling and *Tongxie Yaofang* intervention on the composition of intestinal mucosal microbiota in mice, we conducted statistical analysis on the top 10 species in terms of abundance in each group at the phylum and genus levels, and presented the analysis results in a bar chart.

#### 3.3.1 Intestinal mucosal microbiota composition characteristics at the phylum level

**Figure 4:**
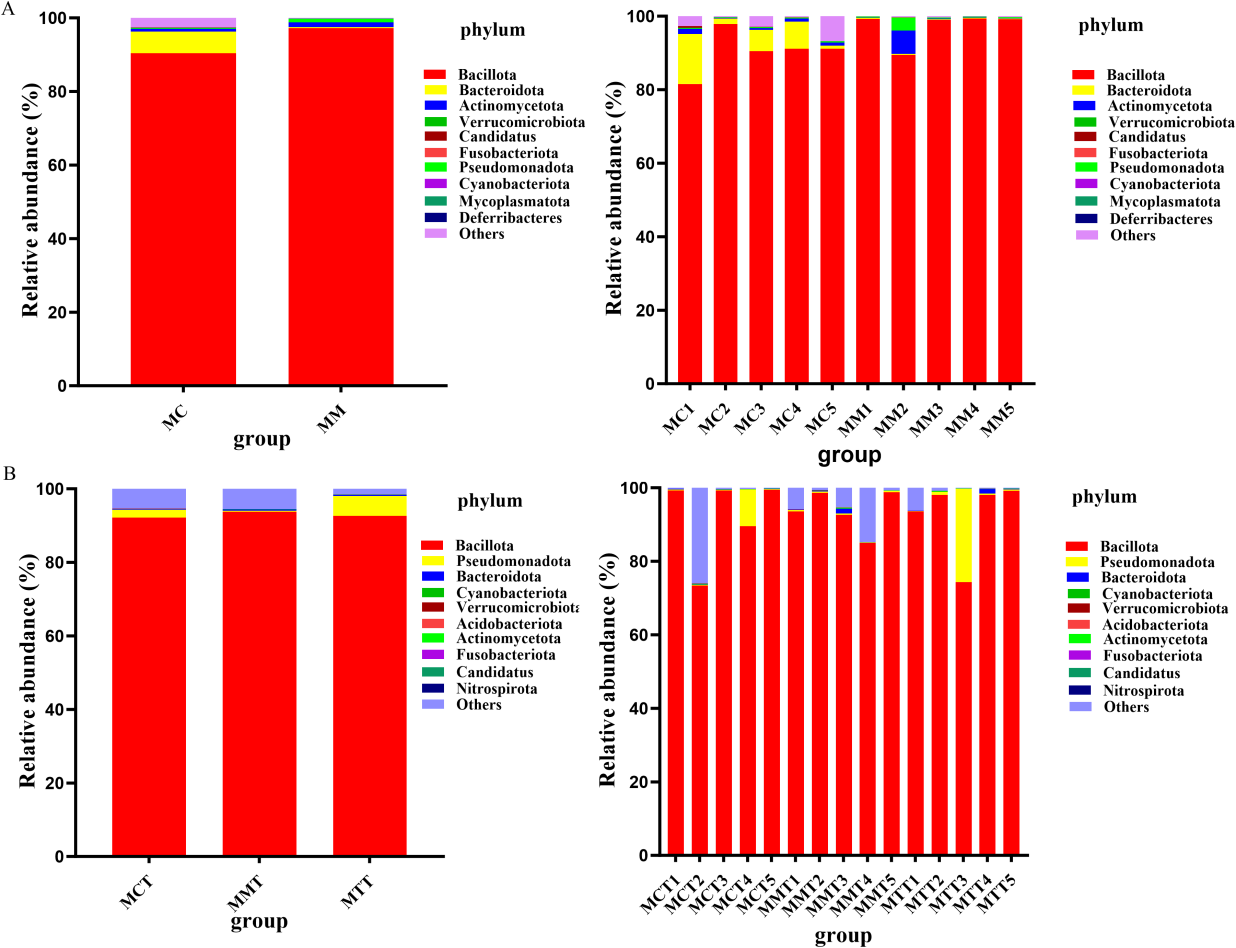
The dominant microbiota of the intestinal mucosa in mice at the phylum level. **(A)** modeling experiment; **(B)** treatment experiment. MC stands for the control group, MM stands for the model group; MCT stands for the control group, MMT stands for the natural recovery group, MTT stands for the treatment group.

The composition and abundance distribution of the samples can be obtained at the phylum level based the QIIME software. Except for unclassified microbiota, our research results indicated that Bacillota was the phylum of intestinal mucosal microbiota in mice and held an absolute position. Specifically, it was as follows (**Figure 4**):

In the modeling experiment, the distribution proportion of Bacillota in MC has reached 90.43%, followed by Bacteroidota (5.84%), Actinomycetota (2.56%), and Pseudomonadota (0.27%). In MM, the distribution proportion of Bacillota was higher, accounting for 97.23%, next to Bacteroidota (0.23%), Actinomycetota (0.21%), and Pseudomonadota (0.90%).

In the treatment experiment, we obtained the same conclusion. The dominant phyla in MCT, MMT and MTT samples were Bacillota (92.19%, 93.70% and 92.64% respectively), Bacteroides (0.12%, 0.34% and 0.29% respectively). As showed in results, although there were slight differences in the relative abundance among the three groups at the same microbiota level, there was no statistical significance.

The above results indicated that the abundance difference mainly came from Bacillota. The modeling of *Ganqichengpi* syndrome diarrhea increased the relative abundance of Bacillota, significantly raised the ratio of Bacillota to Bacteroides. After treatment with *Tongxie Yaofang* prescription, it basically returned to the normal group level.

#### 3.3.2 Intestinal mucosal microbiota composition characteristics at the genus level

**Figure 5:**
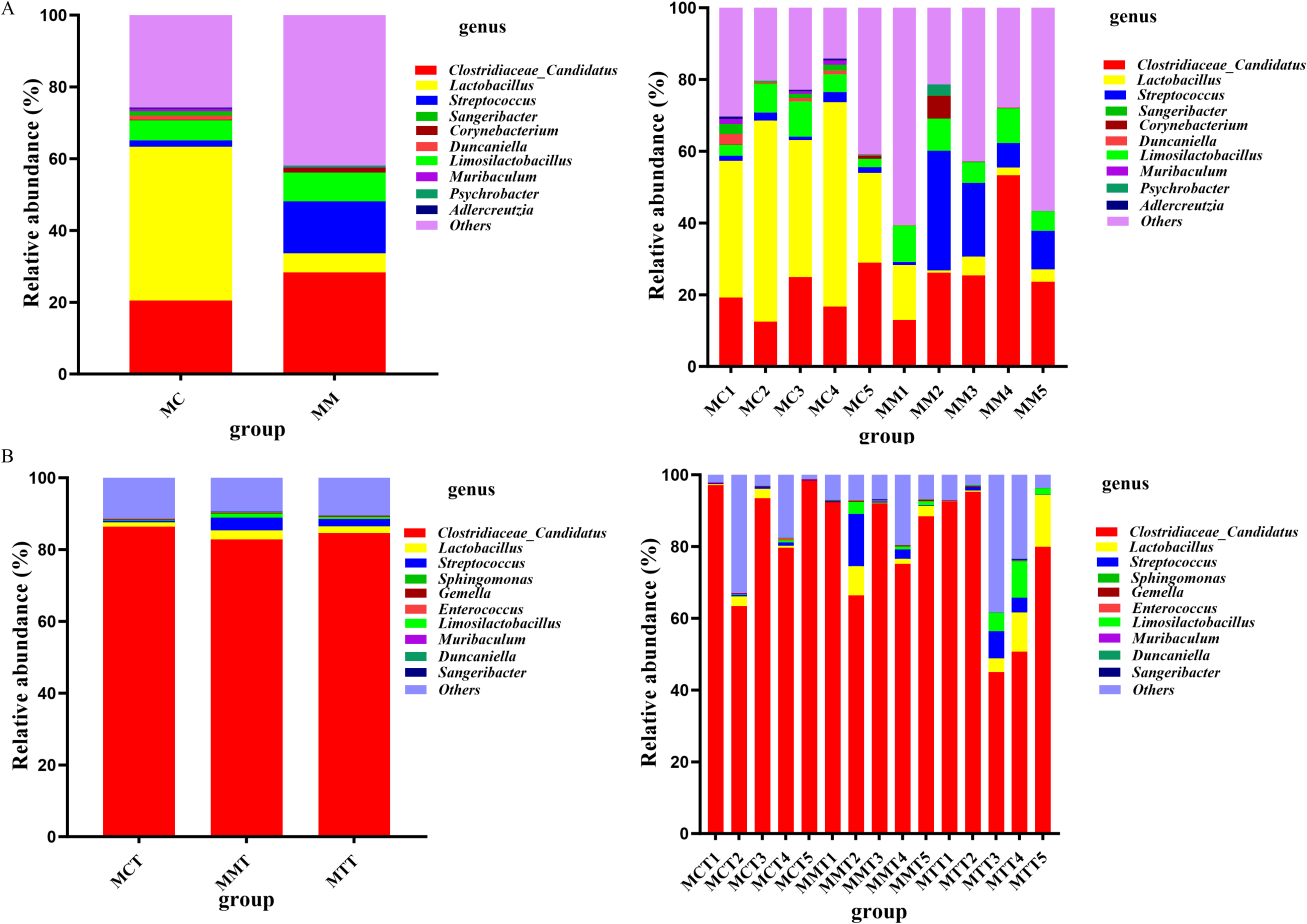
The dominant microbiota of the intestinal mucosa in mice at the genus level. **(A)** modeling experiment; **(B)** treatment experiment.

In the modeling experiment, a total of 213 genera were detected in 10 samples of the two groups. 161 and 153 genera were detected in MC and MM, respectively. Among the identified genera, the top three genera in relative abundance were *Clostridiaceae Candidatus*, *Lactobacillus*, and *Streptococcus* (**Figure 5**). Among them, there was a significant difference between the two groups in *Lactobacillus* (*P* = 0.002). Furthermore, compared with MC samples, the relative abundance of *Clostridiaceae Candidatus* and *Streptococcus* increased in MM samples. However, the relative abundance of *Lactobacillus* was significantly reduced in MM samples. In addition, the number of unclassified microbiota genera increased significantly in MM samples.

In the treatment experiment, 217 genera were detected in 15 samples of the three groups. 119, 138 and 121 genera were detected in MCT, MMT and MTT, respectively. Among the detected genera, except for unclassified microbiota, the top three genera in relative abundance remained *Clostridiaceae_Candidatus*, *Lactobacillus* and *Streptococcus* (**Figure 5**). After treatment with *Tongxie Yaofang* prescription, the relative abundance of *Lactobacillus* increased, while the relative abundance of *Clostridiaceae_Candidatus* and *Streptococcus* decreased. Furthermore, the abundance of unclassified genera in MTT samples was close to those in MCT samples.

### 3.4 The effect of *Ganqichengpi* syndrome diarrhea and *Tongxie Yaofang* intervention on intestinal mucosal microbiota in differential genera

**Figure 6:**
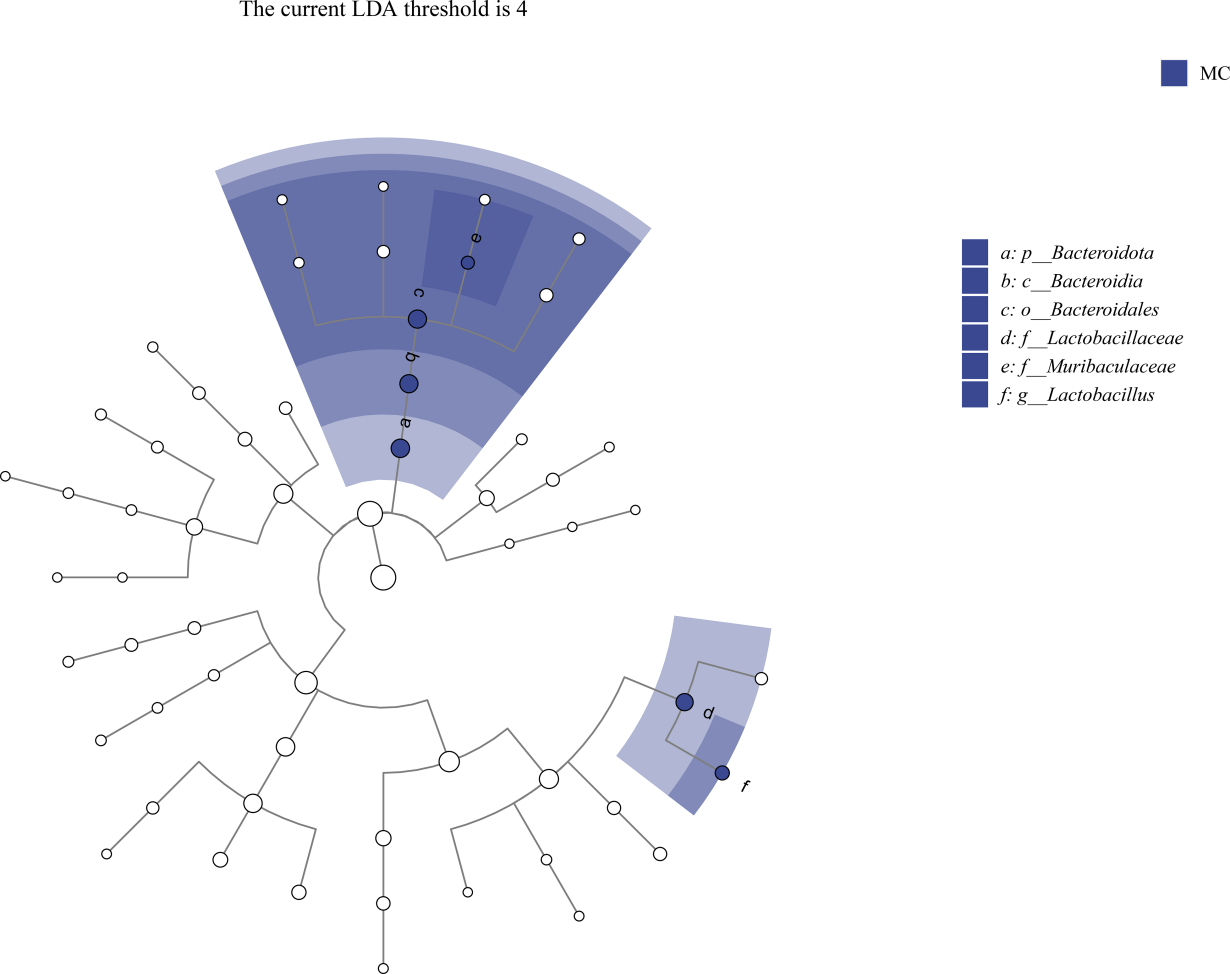
The differential microbiota of the intestinal mucosa in mice. MC: the control group.

To determine the species with significant differences in abundance among each group, LEfSe analysis was conducted on the composition of intestinal mucosal microbiota in each group. As seen in **Figure 6**, in the modeling experiment, *Lactobacillus* was the biomarker genus in MC samples with a logarithmic LDA threshold of 4.0. However, in the treatment experiment, under the same LDA threshold conditions, no biomarker genera in each group were found.

### 3.5 Predictive analysis of intestinal mucosal microbiota function

**Figure 7:**
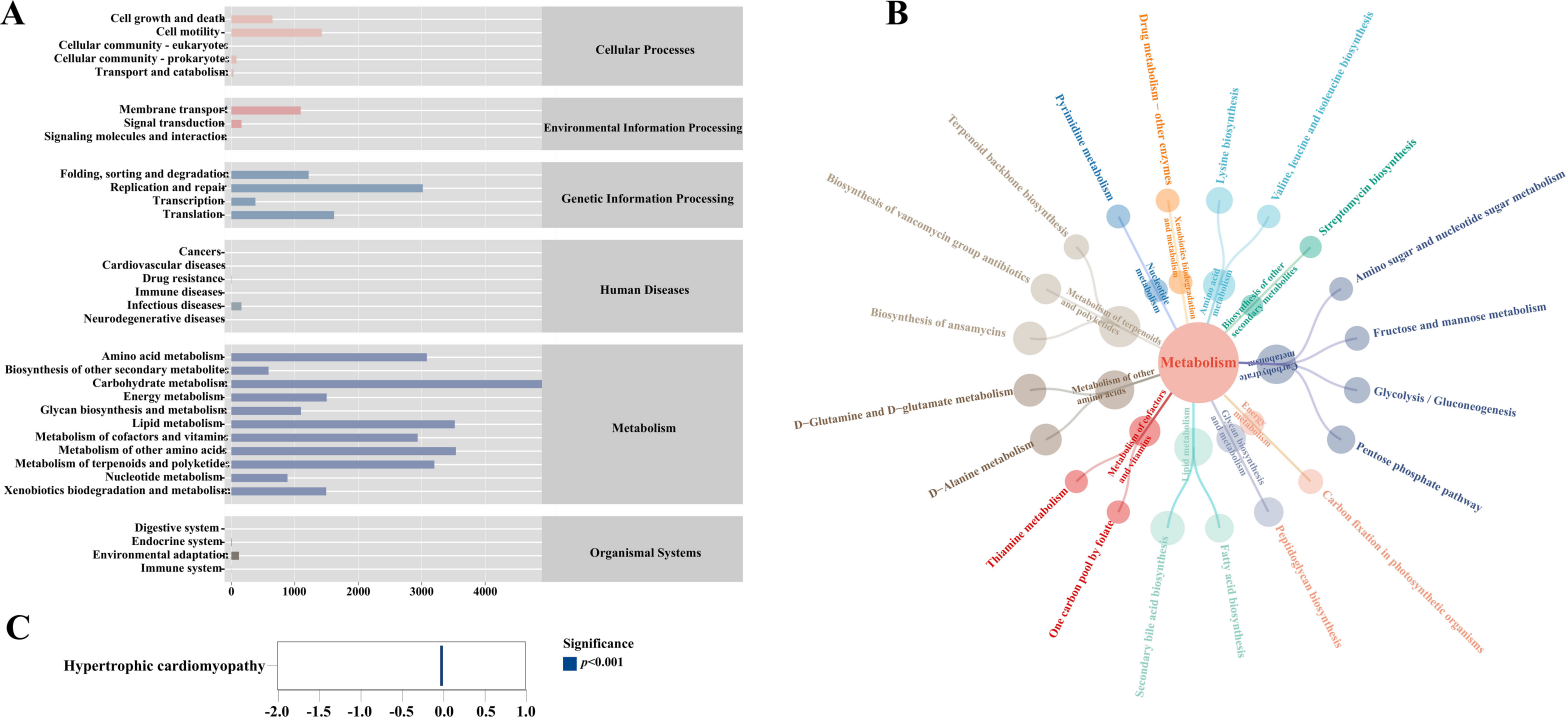
KEGG functional analysis of intestinal mucosal flora in mice. **(A)** Functional prediction abundance map (first-level and second-level); **(B)** Three-layer dependency network diagram; **(C)** Analysis of Metabolic Pathway Differences (MMT *vs* MTT).

PICRUSt 2 was used to analyze and predict the KEGG signaling pathway of intestinal mucosal microbiota. As shown in **Figure 7A**, modeling and drug intervention had the greatest impact on metabolic function. Among them, the top 20 pathways in terms of metabolic pathway abundance were Secondary bile acid biosynthesis, Biosynthesis of ansamycins, D-Glutamine and D-glutamate metabolism, D-Alanine metabolism, Biosynthesis of vancomycin group antibiotics, Peptidoglycan biosynthesis, Fatty acid biosynthesis, Pentose phosphate pathway, Lysine biosynthesis, Glycolysis/Gluconeogenesis, Terpenoid backbone biosynthesis, Carbon fixation in photosynthetic organisms, Fructose and mannose metabolism, Pyrimidine metabolism, Amino sugar and nucleotide sugar metabolism, Drug metabolism - other enzymes, Valine, leucine and isoleucine biosynthesis, One carbon pool by folate, Thiamine metabolism, Streptomycin biosynthesis, and their subordinate relationships were shown in **Figure 7B**. The results of the analysis of metabolic pathway differences showed that compared with the MMT group, the abundance of Hypertrophic cardiomyopathy in the MTT group decreased significantly (*p* < 0.01; **Figure 7C**).

### 3.6 Comparison of 5-HT, VIP and BDNF levels in serum

**Figure 8:**
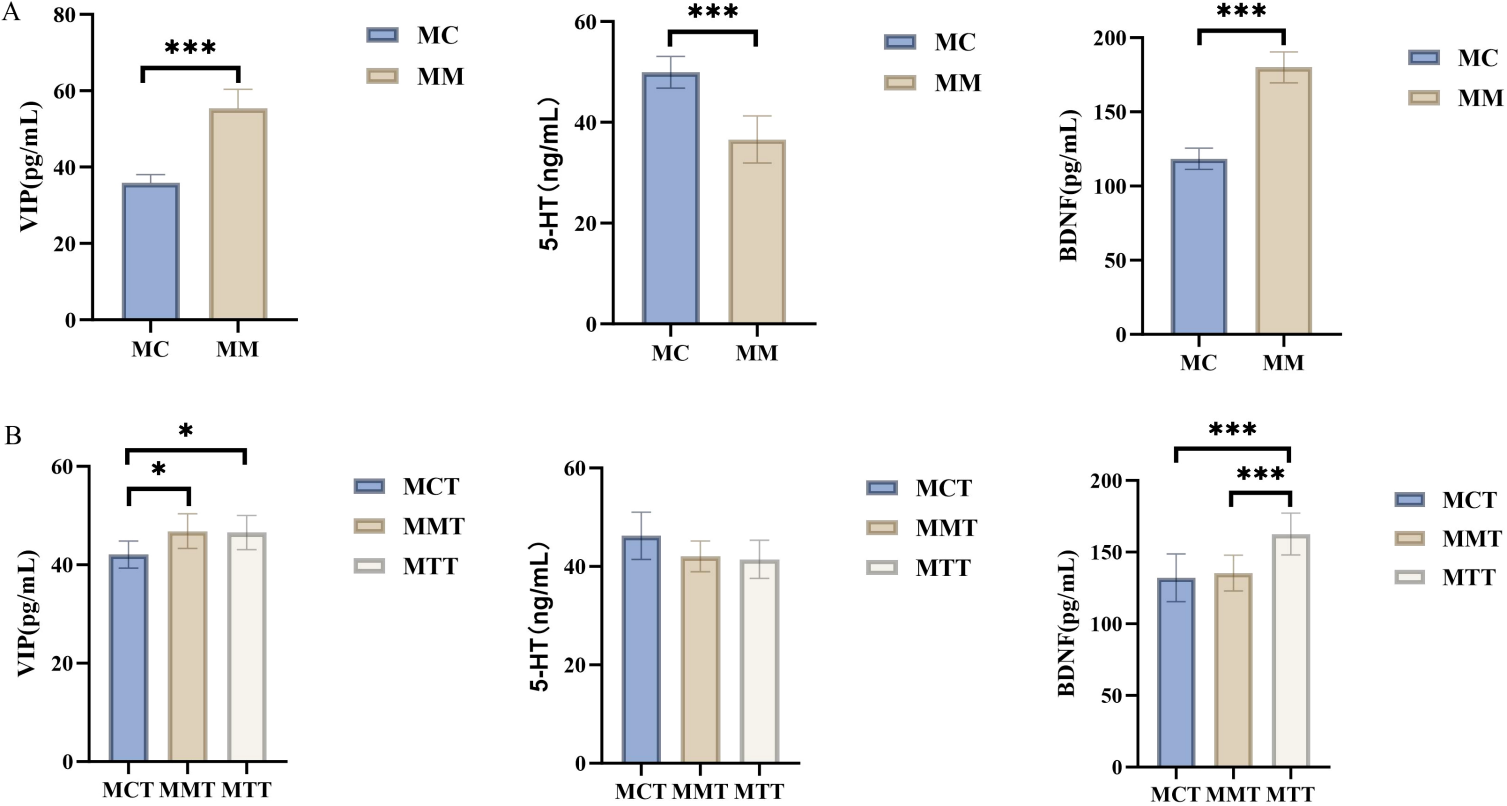
Comparison of 5-HT, VIP and BDNF levels in serum. **(A)** modeling experiment; **(B)** treatment experiment. MC stands for the control group, MM stands for the model group; MCT stands for the control group, MMT stands for the natural recovery group, MTT stands for the treatment group." and “*stands for p<0.05,***stands for p<0.01.

In the modeling experiment, VIP and BDNF in the serum of mice in the model group significantly increased, while 5-HT significantly decreased (*p* < 0.01; **Figure 8A**). In the treatment experiment, the serum VIP level of mice in the drug intervention group showed a decreasing trend, but there was still a statistically significant difference compared with the normal group (*p* < 0.05; **Figure 8B**). Compared with the normal group, the serum 5-HT levels of mice in the natural recovery group and the drug intervention group decreased, and there was no statistical difference among the three groups. The serum BDNF level in the natural recovery group of mice decreased, with no significant difference from that in the normal group. However, the BDNF level in the drug intervention group was significantly higher than that in the natural recovery group and the normal group (*p* < 0.01). The results suggested that the drugs used in the treatment group had no significant effect on serum 5-HT and VIP, and inhibited the reduction of BDNF.

## 4 Discussion

The intestinal microbiota is an important component of the intestinal microecology, participating in material digestion, energy metabolism, nutrient absorption, immune regulation and mucosal barrier, etc., and playing an important role in the human health (Long et al., 2020). Under normal circumstances, the intestinal microbiota is in a relatively stable dynamic balance. Once this balance is disrupted, disorders will occur in the types, quantities, proportions, and locations of the intestinal microbiota, resulting in intestinal dysbacteriosis (Parker et al., 2018). The changes of certain specific microbiota in the body may become the main cause of the occurrence and development in certain diseases (Long et al., 2023). In this study, we selected the intestinal mucosal environment with relatively stable microbiota colonization and used the second-generation high-throughput sequencing technology to explore the effect of *Tongxie Yaofang* prescription on the intestinal mucosal microbiota in mice with *Ganqichengpi* syndrome diarrhea. These research results will provide experimental support for the microecological mechanism of traditional Chinese medicine prescription in the treatment of diarrhea and related intestinal diseases.

### 4.1 *Tongxie Yaofang* prescription demonstrated potential in restoring intestinal mucosal microbiota diversity, although its efficacy remained relatively limited

In recent years, investigating the correlation between intestinal diseases and microbiota from the perspective of bacterial diversity has emerged as a significant research focus (Long et al., 2020). The modeling experiments demonstrated that *Ganqichengpi* syndrome diarrhea modeling decreased the diversity of intestinal mucosal microbiota in mice and markedly increased the types of unclassified genera, which was consistent with the effect of *Ganqichengpi* syndrome diarrhea modeling on the intestinal contents microbiota in mice (Liu, 2022). However, in the treatment experiment, the values of each alpha diversity index in MMT were slightly higher than those in MCT. The possible reason could be attributed to the robust self-regulating capacity of the mucosal microbiota in mice, which exhibited a strong recovery potential. Following short-term intervention through modeling, the microbiota was able to restoration naturally (Long et al., 2020). After treatment with *Tongxie Yaofang* prescription, the Chao1 index and Observed species returned to MCT level, while the Simpson and Shannon indices increased, but they were not statistically significant. In particular, the treatment with *Tongxie Yaofang* prescription significantly reduced the microbiota species of unclassified genera, approaching the level in MCT samples. The results indicated that *Tongxie Yaofang* has a certain promoting effect on intestinal mucosal microbiota diversity, although the efficacy remained relatively limited.

### 4.2 *Tongxie Yaofang* prescription changed the community structure in intestinal mucosal microbiota, but the effect was relatively subtle

Overall, whether it was PCoA or NMDS, the distances between different samples were relatively discrete, indicating that the similarity of communities was not high and there were significant differences. In the modeling experiment, the samples in MM were more dispersed than those in MC, indicating that the modeling had an impact on the community structure of intestinal mucosal microbiota. In the treatment experiment, the samples within MCT and MMT were relatively concentrated, while the samples within MTT were relatively discrete, indicating that there were significant differences within the groups. This result was consistent with the conclusion drawn from our previous research on the intestinal contents (Liu, 2022). The possible reasons might be the individual differences among the mice themselves, as well as variations in the sensitivity of different mice within the group to the modeling methods and therapeutic drugs. From the composition characteristics of OTUs and unclassified microbiota, we could draw the same conclusion.

### 4.3 *Tongxie Yaofang* prescription effectively restored the abundance of intestinal mucosal microbiota, with significant alterations in the dominant microbiota

The existence of the dominant microbiota demonstrates the significance of the host in selecting the composition of the microbial community and plays a crucial role in maintaining intestinal function and health (Wu et al., 2025b). In this study, Bacillota was the dominant phylum in the intestinal mucosal microbiota of mice, followed by Bacteroidota, Actinomycetota and Pseudomonadota. Bacillota and Bacteroidetes are regarded as important phyla involved in energy absorption, protein synthesis and lipid metabolism (Liu et al., 2022; Yan et al., 2025). The ratio of Bacillota to Bacteroidetes is an important indicator in reflecting the organism health and reveals the state of intestinal microecological balance (Zhou, 2023). In this study, *Ganqichengpi* diarrhea modeling led to a significant increase in the relative abundance of Bacillota and a significant decrease in Bacteroidetes, causing an abnormal change in the ratio of Bacillota to Bacteroidetes. After treatment with *Tongxie Yaofang* prescription, their abundance returned to MCT level. These changes in results indicated that *Tongxie Yaofang* prescription reduced the ratio of Bacillota to Bacteroidetes, promoted the composition and abundance of beneficial microbiota such as Bacteroidetes, and effectively restored the abundance of intestinal mucosal microbiota.

The differences at the phylum levels usually depend on specific variations at the genus and species levels. At the genus level, *Clostridiaceae_Candidatus*, *Lactobacillus*, *Streptococcus* and *Limosilactobacillus* were the main genera of the intestinal mucosa in mice. These results were consistent with intestinal contents (Liu, 2022). In this study, *Ganqichengpi* syndrome diarrhea modeling led to an increase in the relative abundance of *Clostridiaceae_Candidatus* and a significant decrease of *Lactobacillus*. After the treatment with *Tongxie Yaofang* prescription, the relative abundance of *Clostridiaceae candidatus* decreased, while the relative abundance of *Lactobacillus* significantly increased. The relative abundance of *Lactobacillus* was the highest in MTT samples and the lowest in MCT samples. These results were consistent with the microbiota composition of intestinal contents in mice (Liu, 2022). LEfSe analysis further indicated that *Lactobacillus* was the biomarker genus in MM in the modeling experiment. However, under the premise of a threshold of 4.0, no biomarker genus was found in the treatment experiment.

### 4.4 *Tongxie Yaofang* prescription has no significant effect on 5-HT and VIP, but it inhibits the reduction of BDNF

5-HT, also known as serotonin, was a relatively crucial neurotransmitter in the central nervous system (Iwasaki et al., 2019). It can bring about pleasant emotions and plays a crucial role in regulating one’s mental state. The disorder of the concentration and functional activity of 5-HT was closely related to the onset of various affective disorders. The reduction of its concentration and function was an important physiological and pathological basis for the generation and development of depression. People with lower levels of 5-HT were more prone to depression, aggression and violent behavior (Zang, 2020). This experimental study found that the 5-HT level in the model group mice was significantly lower than that in the normal group, indicating that the decrease in 5-HT level is one of the pathogenic mechanisms of *Ganqichengpi* diarrhea. This is consistent with the symptoms of irritability, anger, and a tendency to fight in the model group mice (Liu et al., 2020). In the treatment experiment, there was no statistically significant difference in the level of 5-HT in the serum of mice among the groups. Combined with the characteristic manifestations of the model of *Ganqichengpi* syndrome diarrhea, it is speculated that 5-HT may mainly be involved in the occurrence and development of the disease, rather than being the main target of *Tongxie Yaofang* in treating diarrhea. Its regulatory mechanism may involve more complex nervous system regulatory functions.

VIP, also known as vasodilator intestinal peptide, was an important fetal neurotransmitter (Zhang et al., 2023). VIP has diverse functions. It can not only inhibit gastrointestinal motility but also promote the secretion of intestinal water and electrolytes. Clinically, it was associated with various diseases, especially gastrointestinal diseases, and was regarded as one of the important indicators in the research of gastrointestinal diseases (Zang, 2020). The results of this study revealed that modeling significantly increased VIP levels, which was consistent with the physiological role of VIP in promoting intestinal fluid secretion and may be an important mechanism leading to diarrhea. After treatment with the *Tongxie Yaofang*, although the VIP level decreased, there was no statistical difference compared with the natural recovery group. This suggested that the recovery of VIP levels may be more related to the natural outcome of the disease rather than the result of heterogeneous drug regulation. This provides a new experimental basis for clarifying the characteristics of the *Tongxie Yaofang*.

BDNF was a neuropeptide that was crucial for the development and regulation of the central nervous system (Messaoud et al., 2011). Previous studies have shown that the intestinal microbiota was closely related to the central nervous system. The key pathways for communication between the intestinal microbiota and the brain include the vagus nerve, tryptophan metabolites, and microbial metabolites such as exercise fatty acids or peptidoglycan (Yaklai et al., 2021). BDNF, as a brain-derived neurotrophic factor, was closely related to anxiety and depression (Notaras and van den Buuse, 2020; Zhuang et al., 2022). The results of this study showed that modeling significantly increased the level of BDNF, which may reflect the compensatory neuroprotective mechanism of the body under stress conditions. After the intervention of *Tongxie Yaofang*, although the BDNF level decreased, it was still significantly higher than that of the normal group and the natural recovery group. This regulatory pattern suggests that *Tongxie Yaofang* may achieve the therapeutic effect on *Ganqichengpi* syndrome diarrhea by maintaining an appropriate BDNF level, which not only alleviates the excessive stress response under pathological conditions but also retains the necessary neuroprotective effect. This conclusion was also verified in the functional prediction that the abundance of Hypertrophic cardiomyopathy in the MTT group decreased significantly compared with that in the MMT group. It is indicated that the therapeutic effect of *Tongxie Yaofang* in treating diarrhea syndrome caused by *Ganqichengpi* has a certain relationship with the level of BDNF, but its specific mechanism of action awaits further study.

### 4.5 Possible pathways in which *Tongxie Yaofang* prescription regulated intestinal mucosal microbiota structure

The research results indicated that *Tongxie Yaofang* prescription could alter the types, quantities, richness and diversity of intestinal mucosal microbiota. The possible pathways include the following three aspects.

Firstly, the active ingredients in *Tongxie Yaofang* prescription can directly act on the intestinal microbiota, promoting the growth of probiotics while inhibiting pathogenic and opportunistic bacteria, and restoring the balance of the intestinal microecology (Yuan et al., 2020a). Specifically, the polysaccharide substances in *Rhizoma Atractylodis Macrocephalae* and *Radix Paeoniae Alba* can promote the growth of intestinal probiotics (Feng and Wu, 2019). The flavonoids, alkaloids, polysaccharides and their extracts in *Pericarpium Citri Reticulatae* can promote the recovery of probiotics (Chen, 2019). The effective components in the decoction, such as *atractylodes macrocephala*, *paeoniflorin*, *hesperidin*, and *chromogenic ketone*s, can effectively inhibit opportunistic pathogenic bacteria and pathogenic bacteria (Zhang et al., 2019). In addition, *Radix Saposhnikoviae* plays an important role in regulating the intestinal microbiota and treating diarrhea (Liu, 2022).

Secondly, *Tongxie Yaofang* prescription regulates the intestinal microbiota by enriching beneficial bacteria. This includes reducing the ratio of Bacillota to Bacteroidetes to produce beneficial effects by increasing the abundance and quantity of Bacteroidetes in the intestine, or enriching the content of probiotics such as *Lactobacillus* (Liu et al., 2022). In this study, *Ganqichengpi* syndrome diarrhea modeling significantly increased the ratio of Bacillota to Bacteroides. After treatment with *Tongxie Yaofang* prescription, the ratio significantly decreased and returned to MCT level. Furthermore, *Lactobacillus* was significantly enriched in MTT.

Finally, *Tongxie Yaofang* prescription increases the production of short-chain fatty acids (SCFAs) such as acetic acid, propionic acid, and butyric acid by restoring intestinal balance, promoting the growth and metabolic activities of beneficial bacteria, and inhibiting the excessive growth of opportunistic pathogenic bacteria (such as Proteobacteria) (Qin, 2021). SCFAs, especially butyric acid, are the main energy source for colonic epithelial cells. They can promote the proliferation and repair of epithelial cells and enhance the expression of tight junction proteins, thereby strengthening the integrity of the intestinal mucosal barrier (Wu et al., 2025a). This might be one of the important mechanisms of *Tongxie Yaofang* prescription in treating diarrhea.

In conclusion, *Tongxie Yaofang* prescription inhibited the reduction of serum BDNF level in mice with *Ganqichengpi* syndrome diarrhea. The effects on 5-HT and VIP were not significant, and the therapeutic mechanism was not closely related to the regulation of brain-gut peptide function. *Tongxie Yaofang* prescription may treat diarrhea caused by *Ganqichengpi* syndrome diarrhea by regulating dominant bacterial genera, increasing the relative abundance of *Lactobacillus*, reducing the filamentous genus of *Clostridium*, and restoring intestinal balance.

## Data Availability

All raw data within this study has been deposited in the NCBI repository, accession number is PRJNA 1280487. (http://www.ncbi.nlm.nih.gov/bioproject/1280487).
